# Chirality and Numbering of Substituted Tropane Alkaloids

**DOI:** 10.3390/molecules16097199

**Published:** 2011-08-25

**Authors:** Munir Humam, Tarik Shoul, Damien Jeannerat, Orlando Muñoz, Philippe Christen

**Affiliations:** 1 School of Pharmaceutical Sciences, University of Geneva, University of Lausanne, Quai Ernest-Ansermet 30, CH-1211 Geneva 4, Switzerland; Email: munir_humam@hotmail.com (M.H.); t.shoul@hotmail.com (T.S.); 2 Department of Organic Chemistry, University of Geneva, Quai Ernest-Ansermet 30, CH-1211 Geneva 4, Switzerland; Email: damien.jeannerat@unige.ch; 3 Departamento de Química, Facultad de Ciencias, Universidad de Chile, Casilla 653, Santiago, Chile; Email: omunoz@uchile.cl

**Keywords:** tropane alkaloids, *Schizanthus*, molecular configuration, Mosher’s esters, (3*R*,6*R*)-3α-hydroxy-6β-senecioyloxytropane

## Abstract

The strict application of IUPAC rules for the numbering of tropane alkaloids is not always applied by authors and there is hence a lot of confusion in the literature. In most cases, the notation of 3, 6/7-disubstituted derivatives has been chosen arbitrarily, based on NMR and MS data, without taking into account the absolute configuration of these two carbons. This paper discusses the problem and the relevance of CD and NMR to determine molecular configurations. We report on the use of ^1^H-NMR anisochrony (Δδ) induced by the Mosher’s chiral auxiliary reagents (*R*)-(-)- and (*S*)-(+)-α-methoxy-α-trifluoromethyl-phenylacetyl chlorides (MTPA-Cl), to determine the absolute configuration of (3*R*,6*R*)-3α-hydroxy-6β-senecioyloxytropane, a disubstituted tropane alkaloid isolated from the aerial parts of *Schizanthus grahamii* (Solanaceae). These analytical tools should help future works in correctly assigning the configuration of additional 3, 6/7 disubstituted tropane derivatives.

## 1. Introduction

Unambiguous identification and structure elucidation of bioactive natural products is of paramount importance for understanding their physical and chemical properties. While relative configuration of chiral centres can usually be assigned by NMR (NOESY and/or NOE) experiments [1], the determination of the absolute configuration is much more difficult to obtain. The NMR spectra of enantiomers being identical, one has to introduce chirality in the medium to break the symmetry. Among the different possibilities, the use of chiral lanthanide shift reagents (LSI) [2], chiral polymers [3] or chiral ions [4] allows, in the best case, to observe a split between the enantiomer signals. A more reliable method consists in using chiral auxiliary reagents such as Mosher's (*R*)-(-)- and (*S*)-(+)-α-methoxy-α-trifluoromethylphenylacetyl acetic acid (MTPA) combined with NMR measurements. It is often employed for the characterization of various natural products bearing secondary alcohols, amines or carboxylic functions [5,6,7]. Circular dichroism is another technique sensitive to the chirality of molecules, but the correlation with the configuration is not straightforward and has to be applied with some precautions [4]. Only X-ray crystallography [8] provides direct access to the chirality of compounds but obviously relies on the ability to form crystals of the compounds of interest.

**Figure 1:**
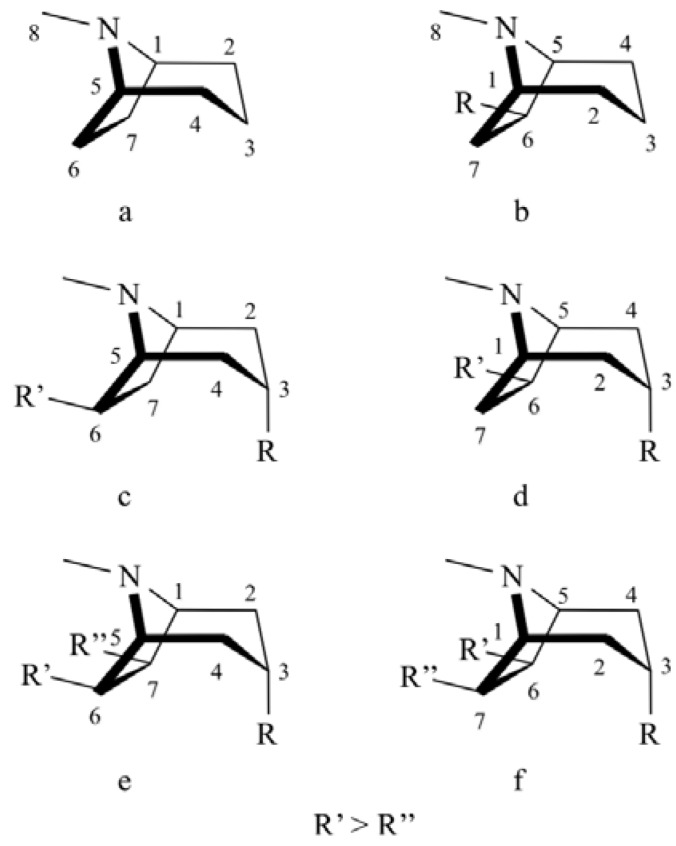
Tropane nucleus numbering, a, c and e: clockwise; b, d and f: anticlockwise.

In many cases, the absolute configuration of isolated compounds is not mentioned in the original articles, and often misleading information is provided either by improper structural drawings or assuming, but not specifying, that the reported compounds have the same configuration as those previously reported. Tropane alkaloids are a well-known and important class of natural products because of their pharmacological activities as anticholinergic agents. They are widely distributed in the Solanaceae, Erythroxylaceae, Convolvulaceae, Proteaceae and Rhizophoraceae plant families. These alkaloids encompass a wide range of mono-, di- and trisubstituted derivatives, having in common the tropane (8-azabicyclo[3.2.1]octane) nucleus as the key structural element (Figure 1a). They are often esterified with various aliphatic and aromatic acids [9].

There is a lot of confusion in the literature about the numbering of the tropane skeleton [9]. The IUPAC recommendations [10] stipulate that the skeleton be numbered clockwise, starting from the C-1 being at the “back” when the C-3 hydroxyl is to the right (Figure 1a). However, depending on the substituent in position C-6/C-7, the C-1 must be at the “front” and the numbering then be anticlockwise so that the carbon bearing the substituent has the lowest possible number (Figure 1b).

Except for 1-hydroxytropane, most of the monosubstituted alkaloids are derived from tropine (3-hydroxytropane) esterified at the C-3 position. The latter, containing chiral centres (C-1 and C-5), is an optically inactive symmetrical molecule and can be regarded as a *meso* compound. Most of tropane alkaloids are either disubstituted in C-3, C-6 (often described in the literature as a C-3, C-7) Figure 1c and 1d) or trisubstituted in C-3, C-6 and C-7 [9,11] (Figure 1e and 1f). In most cases, the notation of 3, 6/7-disubstituted derivatives has been chosen arbitrarily without taking into account the absolute configuration of these two positions. Typical examples are the 3,6/7-disubstituted tropane alkaloids identified by GC-MS only [12,13]. Most of these compounds have been identified under achiral conditions by their retention times, Kovats or Van den Dool indices, fragmentation patterns and sometimes by comparison with reference compounds whose structures have not themselves been unambiguously characterized. None of these methods distinguish the enantiomers.

Within the tropane alkaloid family, many compounds are 3α,6β/7β-tropanediol derivatives that can exist as two stereoisomeric (3*R*,6*R*) or (3*S*,6*S*) species. This stereoisomerism is not well documented and most of these molecules do not have a defined absolute configuration. Only a few compounds have been studied by X-ray diffraction analysis, most of the time in the form of salts [14,15,16]. Frequently, the configuration has been obtained using optical methods and NMR of derivatized compounds.

In this work, we report on the use of NMR spectroscopy using Mosher’s chiral auxiliary reagent to determine the absolute configuration of 3α-senecioyloxy-6β-hydroxytropane (Figure 1c and 1d: R= senecioyloxy; R’=hydroxyl), a typical representative disubstituted alkaloid originating from the genus *Schizanthus*. The method is an alternative to circular dichroism (CD) [17,18,19].

## 2. Determination of the Configuration Using Optical Properties

Regarding stereochemistry, the specific rotation [α] of a compound is usually sufficient to determine its absolute configuration. The sign of the angle is opposite for pairs of enantiomers and allows one to distinguish them. However, for the tropane alkaloids, the sign of the specific rotation is known in only a few cases. This is due, in part, to the difficulty to deduce a rule correlating signs and configurations. Some attempts have been made, but these sometimes have led to missassignments [20], which have been corrected later [21,22]. In principle, the hydrolysis of tropane esters, leading to 3,6/7-tropanediol should solve the problem, because *R,R* enantiomer is levorotatory (−24°) [18,23]. However, this method proved unreliable, probably because of the difficulty to ensure a defined conformation of the OH groups, as the latter are very sensitive to any protonation of the amine and to the presence of water or other hydrogen donors or acceptors in the solvent [24].

In principle the full electronic circular dichroism (eCD) [17,19] spectra of compounds should be more informative, especially when UV-active chromophores are present. The experimental spectra can then be compared to computer simulations based on the 3D structure of the compound. However difficulties arise when the molecules are conformationally labile because it then requires one take into account the conformational space. This has been applied to esters of 3,6/7 tropanes [19] and α,β-conjugated esters [17]. The disadvantage of this method is that it is a computationally demanding task, especially when different conformations have to be Boltzmann weighted. Furthermore, it requires the presence of substituents containing chromophores. Conversely, the main advantage of the method is that it is non-destructive and it opens the possibility of working by analogy with similar compounds as long as the structure and dynamic can be assumed to be very similar.

Vibrational circular dichroism (vCD) spectra [25] are used very similarly to eCD. They should be easier to use because simulations are computationally less demanding than those of eCD spectra and more general, because they do not require UV-active chromophores. Moreover, vibrational spectra have more transitions than electronic spectra, providing more opportunities to observe optical activity. This method has been applied in a few cases, including a disputed case where [α]_D_ was not conclusive [18] and it demonstrated that the methyl esters of 3,6-tropanediols could be reliably used to determine the molecular configuration [26].

The result of this comparison between experimental and simulated CD spectra seems to indicate that diesters are conformationally stable enough to have their relevant structures calculated and can be used for simulation and determination of the molecular configuration. This means that in principle the Kramers-Kronig relation could be exploited to determine the molecular configuration based on simulated eCD and [α]_D_ of compounds. However, only a systematic study of a reasonable number of tropane derivatives and validation with alternative methods can assess whether this will be possible when the computer power limitations will be circumvented.

## 3. Determination of the Configuration Using Mosher’s Reagent

Nuclear magnetic resonance (NMR) is intrinsically insensitive to chirality because enantiomers have identical spectra. The derivatization with chiral auxiliaries produces however changes in the chemical shifts of the neighbouring nuclei (anisotropy) which can be rationalized to determine the absolute configuration. α-Methoxy-α-trifluoromethylphenylacetate (MTPA, or Mosher’s reagent) [27,28,29] is very commonly used and has been applied to the determination of the absolute stereochemistry of disubstituted tropanes [30]. We illustrate here the use of MTPA to determine the molecular configuration of the 3,6-disubstituted tropanes.

**Figure 2:**
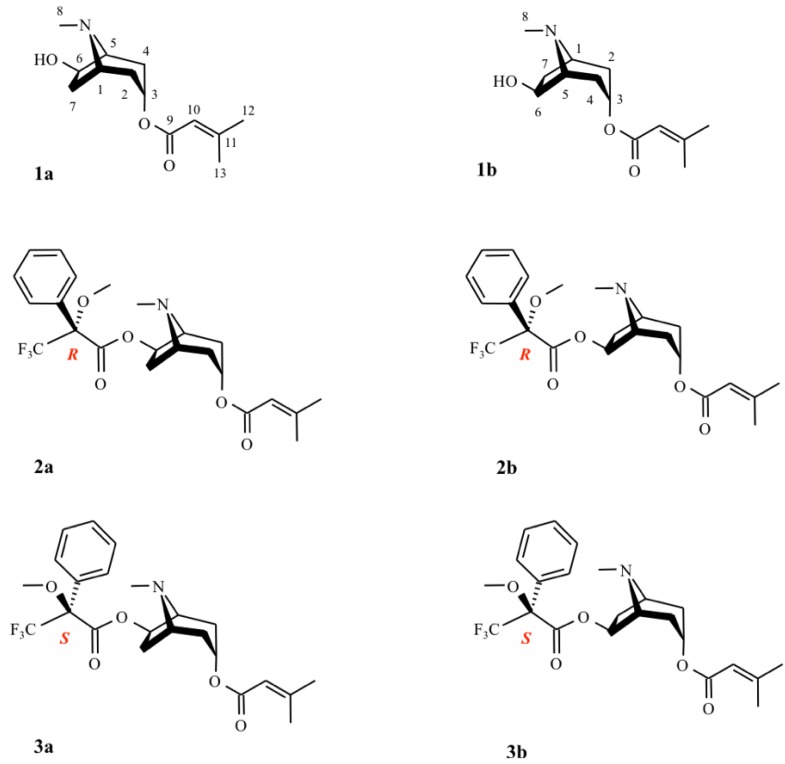
Structures of 3α-senecioyloxy-6β-hydroxytropane **1** and its (*R*)- and (*S*)-Mosher’s esters **2** and **3**.

The absolute configuration of 3α-senecioyloxy-6β-hydroxytropanes **1a** or **1b** (Figure 2), was tentatively determined by circular dichroism (CD). However, because this compound had a very weak Cotton effect, an alternative method was used. Compound **1**was converted into diastereomeric esters **2** and **3** (Figure 2) using (*S*)- and (*R*)-MTPA chlorides, respectively. The absolute configuration of **1** was determined by comparison of the ^1^H-NMR spectra of **2** and **3** according to Dale and Mosher [28].

Comparing to **1**, two different spectra were obtained for these diastereomeric esters (Table 1). We observe that in both esters, all the tropane moiety protons are shielded, except for H-3 which is not modified and H-6, which is the only deshielded proton. Anisochrony (Δδ), expressed as the difference between the chemical shifts of the nuclei affected (Δδ = δ*_R_* − δ*_S_*), is observed for H-5 and H_exo_-7 (+0.1 and −0.12, respectively), and for C-5 and C-7 (−0.4 and +0.7, respectively). Therefore, protons in close proximity to the phenyl group are shielded. As shown in Table 1, the proton at C-7 is shielded in the (*R*)-MTPA derivative, confirming the structure **2a** and excluding structure **2b**. On the other hand, the C-5 proton is shielded in the (*S*)-MTPA derivative, which corresponds to **3a** and not to **3b** (Figure 2). Accordingly, the spectral non-equivalence (Δδ = δ*_R_* − δ*_S_*) between the diastereoisomers gave a positive value for H-5 and a negative value for H*_exo_*-7. This demonstrated unambiguously that the substitution is on C-6 with (*R*) configuration and inducing (*R*) configuration on C-3. Thus, the absolute configuration of 3α-senecioyloxy-6β-hydroxytropane is (3*R*,6*R*).

**Figure 3:**
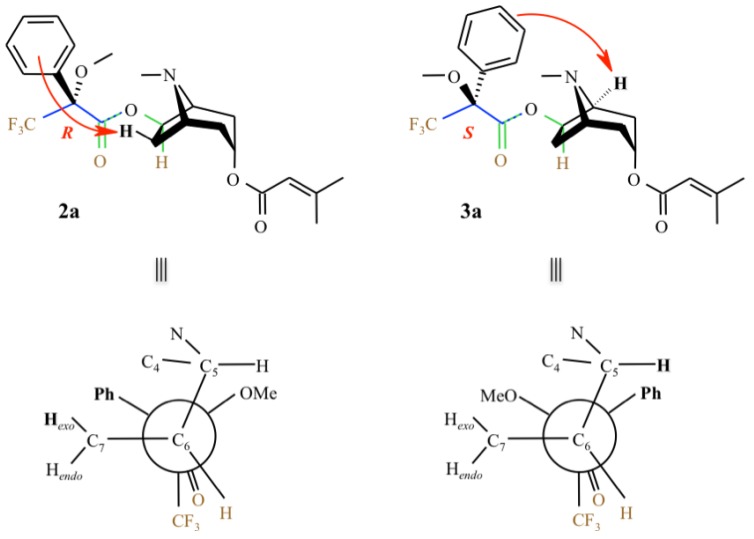
The anisotropy generated by the MTPA’s aromatic ring depends on the configuration and conformation of the auxiliary reagent. To have a significant effect, the phenyl group must produce a direct influence and thus be in a rigid conformation. Earlier experimental [5,27,28,29,31] and theoretical [32] studies showed that the ester group is oriented *anti*-periplanar relative to the CF_3_ group, and *syn*-periplanar with respect to the proton of the chiral carbon. The ester, the CF_3_ groups as well as the C-H bond of the chiral carbon are coplanar which makes this part of the molecule rigid.

The result obtained herein was in-line with that previously reported by CD applied to a disubstituted alkaloid [(3*R*,6*R*)-3α-*trans*-hydroxysenecioyloxy-6β-senecioyloxytropane] isolated from the aerial parts of Schizanthus tricolor [17]. The latter, together with **1** and three other isomers isolated from S. grahamii [33,34], all present a negative [α]_D_. In the absence of chiral carbon(*s*) on the lateral chain(*s*), this result suggests that the absolute configuration of tropane alkaloids with a negative [α]_D_ have a (*R*)-configuration.Figure 3

**Table 1:** ^1^H- and ^13^C-NMR data of compounds **1a**, **2a** and **3a** in CDCl_3_ (500 MHz, *δ* in ppm, *J* in Hz).

	**1a**		**2a** **(**(*R*)-MTPA ester)		**3a** **(**(*S*)-MTPA ester)	
	*d *(H)	*d *(C)	*d *(H)	*d *(C)	*d *(H)	*d *(C)
H–1	3.79 (*s*)	61.0	3.32 (br *s*)	60.4	3.31 (br *s*)	60.8 ****
H *endo*–2	2.56-2.45 (*m*)	32.1	2.14-2.13 (*m*)	35.3	2.14-2.13 (*m*)	35.8
H *exo*–2	1.88 (*d*) (*J2exo,2endo* = 15)		1.71-1.67 (*m*)		1.72-1.69 (*m*)	
H–3	5.05 (*s*)	63.0	5.05 (*t*) (*J* = 4.85)	65.5	5.05 (*t*) (*J* = 4.96)	65.5
H *endo*–4	2.56-2.45 (*m*)	30.6	2.20-2.19 (*m*)	34.0	2.21-2.20 (*m*)	34.4
H *exo*–4	2.03 (*d)* (*J4exo,4endo* = 15)		1.93-1.91 (*m*)	**	1.97-1.95 (*m*)	**
H–5	3.67 (*s*)	68.9	3.24 (*s*)	66.1	3.14 (*s*)	66.4
H–6	4.80 (*dd*) (*J6,7exo* = 5)	72.6	5.73 (*dd*) (*J6,7endo* = 7.48, *J6,7exo* = 2.97)	82.0	5.76 (*dd*) (*J6,7endo* = 7.57, *J6,7exo* = 2.95)	82.0
H *endo*–7	2.85 (*dd)* (*J7endo,7exo* = 10, *J7endo,6* = 5)	37.5	2.65 (*dd*) (*J7endo,7exo* = 14, * J7endo,6* = 7.5)	35.5	2.63 (*dd*,) (*J7endo,7exo* = 14, *J7endo,6* = 7.9)	34.8
H *exo*–7	2.30-2.25 (*m*)		2.12-2.09 (*m*)		2.24 (br *s*)	
H3C–N	2.90 (*s*)	37.5	2.34 (*s*)	40.5	2.24 (*s*)	40.8
H–10	5.65 (*s*)	115.6	5.71 (*t*)	116.1	5.70 (*s*)	116.0
			(*4J* = 1.29)			
H–12	2.19 (*s*)	20.3	2.20 (*s*)	20.3	2.20 (*s*)	20.3
H–13	1.93 (*s*)	27.4	1.94 (*s*)	27.5	1.94 (*s*)	27.4
H3C–O			3.56 (*s*)	55.4	3.58 (*s*)	55.4
H *ortho*–Ph			7.55-7.53 (*m*)	127.4	7.55-7.53 (*m*)	127.2
H *meta*–Ph			7.43-7.41 (*m*)	128.5	7.43-7.41 (*m*)	128.4
H *para*–Ph			7.43-7.41 (*m*)	129.7	7.43-7.41 (*m*)	129.6

## 4. Experimental

### 4.1. Plant Material

*Schizanthus grahamii* Gill. was collected in Rengo (Central Chile) and authenticated by Professor Fernanda Pérez (Departamento de Botánica, Facultad de Ciencias, Universidad de Chile); a voucher specimen (no. 22234) has been deposited in the Faculty of Chemistry at the same University. The stem-bark (2.6 kg) was extracted with ethanol (4 × 3.5 L) at room temperature and the filtered alcoholic solution was evaporated to dryness. The residue was taken up in 0.1 M HCl and washed with dichloromethane. The aqueous solution was made alkaline to pH 12 with NH_4_OH and further extracted with dichloromethane yielding a gummy alkaline residue (6.6 g). Further purification on an aluminium oxide column was performed according to Muñoz *et al.* [35], leading to a purified fraction containing compound **1**, together with three other isomers. These four isomers, present also in other species of the genus [36], were isolated as described by Bieri *et al.* [33].

### 4.2. Chemical and Reagents

Ethyl acetate, dichloromethane, chloroform, pyridine, NaHCO_3_, HCl, (*R*)-(-)- and (*S*)-(+)-MTPA-Cl were purchased from Fluka (Buchs, Switzerland).

### 4.3. Apparatus

^1^H- and ^13^C-NMR spectra were recorded in CDCl_3_ using a 500 MHz Bruker DRX instrument (Bruker, Dübendorf, Switzerland) equipped with a QNP probehead. Chemical shift values (*δ*) are reported in parts per million ppm related to tetramethylsilane used as internal standard and coupling constants (*J*) are in Hertz.

### 4.4. Preparation of Mosher’s Esters

3α-Senecioyloxy-6β-hydroxytropane **1** (5.5 mg in 2 mL dichloromethane) was treated with pyridine (0.2 mL) and (*S*)-(+)-α-methyl-α-trifluoromethylphenylacetic acid chloride [(*S*)-(+)-MTPA-Cl, 100 mg]. The mixture was stirred at room temperature under an atmosphere of nitrogen for 6 h. The reaction mixture was then dried using N_2_ speed-vacuum. The residue was dissolved in a solution of 10% HCl and washed with ethyl acetate (3 × 20 mL). The aqueous phase was made alkaline with a saturated solution of NaHCO_3 _and extracted with CHCl_3_ (3 × 20 mL). The organic layer was separated and the solvent was evaporated to give (*R*)-Mosher’s ester derivative **2** (1.5 mg, 15% yield). The (*S*)-Mosher’s ester derivative **3** (1.5 mg, 15% yield) was prepared using (*R*)-(-)-MTPA-Cl under the same conditions described above.

## 5. Conclusions

The absolute configurations of naturally-occurring tropane alkaloids should be determined by NMR and/or CD whenever they cannot be reasonably assumed to be identical to those of previously determined compounds. In any case, it should be clearly stated if the chirality has been determined and specified. Even if [α]_D_ cannot be used to reliably determine absolute configuration, it should always be reported in order to permit future comparison with experimental and simulated data. Numbering of this kind of molecules should always be established according to their absolute configuration and defined as 3,6-disubstituted compounds.
